# Cardiac injury of the newborn mammalian heart accelerates cardiomyocyte terminal differentiation

**DOI:** 10.1038/s41598-017-08947-2

**Published:** 2017-08-21

**Authors:** David C. Zebrowski, Charlotte H. Jensen, Robert Becker, Fulvia Ferrazzi, Christina Baun, Svend Hvidsten, Søren P. Sheikh, Brian D. Polizzotti, Ditte C. Andersen, Felix B. Engel

**Affiliations:** 1 Experimental Renal and Cardiovascular Research, Department of Nephropathology, Institute of Pathology, Friedrich-Alexander-Universität Erlangen-Nürnberg (FAU), Schwabachanlage 12, 91054 Erlangen, Germany; 2 Laboratory of Molecular and Cellular Cardiology, Department of Clinical Biochemistry and Pharmacology (Odense University Hospital), Winsloewparken 213rd, 5000 Odense C, Denmark; 3 The Danish Regenerative Center (danishcrm.com); Odense University Hospital, Sdr. Boulevard 29, 5000 Odense C, Denmark; 4 Institute of Human Genetics, Friedrich-Alexander-Universität Erlangen-Nürnberg (FAU), Schwabachanlage 10, 91054 Erlangen, Germany; 5 Department of Nuclear Medicine, Odense University Hospital, Odense, Denmark; 6 Institute of Molecular Medicine/University of Southern Denmark, 5000 Odense C, Denmark; 7 Heart Center Translational Research Laboratory, Department of Cardiology, Boston Children’s Hospital, Boston, MA 02115 USA; 8 Department of Pediatrics, Harvard Medical School, Boston, MA 02115 USA; 9 Clinical Institute/University of Southern Denmark, 5000 Odense C, Denmark; 10 Muscle Research Center Erlangen (MURCE), Erlangen, Germany

## Abstract

After birth cardiomyocytes undergo terminal differentiation, characterized by binucleation and centrosome disassembly, rendering the heart unable to regenerate. Yet, it has been suggested that newborn mammals regenerate their hearts after apical resection by cardiomyocyte proliferation. Thus, we tested the hypothesis that apical resection either inhibits, delays, or reverses cardiomyocyte centrosome disassembly and binucleation. Our data show that apical resection rather transiently accelerates centrosome disassembly as well as the rate of binucleation. Consistent with the nearly 2-fold increased rate of binucleation there was a nearly 2-fold increase in the number of cardiomyocytes in mitosis indicating that the majority of injury-induced cardiomyocyte cell cycle activity results in binucleation, not proliferation. Concurrently, cardiomyocytes undergoing cytokinesis from embryonic hearts exhibited midbody formation consistent with successful abscission, whereas those from 3 day-old cardiomyocytes after apical resection exhibited midbody formation consistent with abscission failure. Lastly, injured hearts failed to fully regenerate as evidenced by persistent scarring and reduced wall motion. Collectively, these data suggest that should a regenerative program exist in the newborn mammalian heart, it is quickly curtailed by developmental mechanisms that render cardiomyocytes post-mitotic.

## Introduction

In many instances, regeneration after injury requires remaining cells to undergo varying degrees of dedifferentiation in order to achieve a proliferative state. Dedifferentiation can be defined as a reversal to a progenitor cell-type or appearance of cellular features consistent with, or required for, proliferative competence^1–3^. In cases where a tissue is populated largely by a terminally differentiated cell-type (i.e. one that cannot proliferate or dedifferentiate), regeneration is, at best, limited.

Mammalian cardiomyocytes become terminally differentiated shortly after birth. In brief, within the first three days of birth, rat cardiomyocytes proliferate, increasing in number by 68%^4^. Thereafter, over the ensuing nine days, ~90% of cardiomyocytes re-enter the cell cycle and, rather than divide, binucleate due to failure in completing abscission^4–6^. After this period, cardiomyocytes (mono- and binucleated alike) rarely re-enter or progress through the cell cycle, even after injury^7, 8^. Thus, entry into the binucleation program reflects loss of proliferative competence. The mechanisms that underlie terminal differentiation have not been completely elucidated. However, we have recently shown that, shortly after birth, mammalian cardiomyocytes disassemble their centrosomes – an organelle required for proliferation of mammalian cells –which is coupled with relocalization of various centrosome proteins such as Pericentrin to the nuclear envelope^9^.

The observation that one day-old murine cardiomyocytes are mononucleated and can proliferate has generated great interest in the regenerative potential of the neonatal heart. Initial reports suggested that regeneration after apical resection can occur and is accomplished by global cardiomyocyte proliferation^10^. However, regenerative capacity is greatly diminished when AR is conducted in 1 week-old mice, in agreement with the fact that nearly all cardiomyocytes have attained a terminally differentiated state (i.e. post-mitotic) by this time^10^.

Given that cardiomyocyte proliferative potential is lost by ~P3 in rats and mice during normal neonatal development^4, 6^, extensive regeneration of the newborn heart would require that injury results in a prolonged temporal window of cardiomyocyte proliferative potential (i.e. beyond P3). In this regard, if extensive proliferation-based regeneration occurs, then terminal differentiation would have to be inhibited, delayed, or reversed. Here we tested this hypothesis by analyzing cardiomyocyte centrosome disassembly and binucleation, as markers of terminal differentiation, following apical resection in one day-old murine hearts.

## Results

### Apical resection does not perturb centrosome disassembly

**Figure 1:**
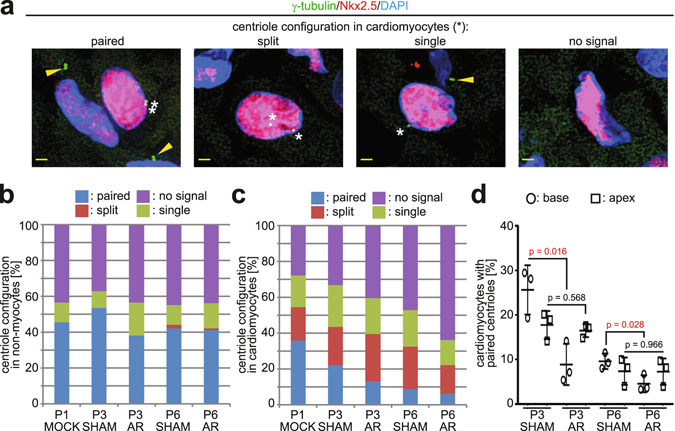
Effect of AR on cardiomyocyte centrosome disassembly. (**a**) Representative images of centrioles (γ-tubulin) in heart cryosections of P0 rat heart ventricles. Nuclei: DAPI. Cardiac nuclei: Nkx2.5. Arrowheads indicate paired centrioles in non-myocytes. Asterisk indicates centrioles in cardiomyocytes. Paired-centrioles: doublet γ-tubulin signals within 2 μm of one another; split-centrioles: γ-tubulin signals greater than 2 μm of one another; single-centriole: single γ-tubulin signal with no identifiable pair (e.g. other centriole is either overlapping or split to the extent of no longer residing in the section); no-centriole: no identifiable, nuclear-proximal, γ-tubulin signal in the section. Scale bars: 2 μm. (**b**,**c**) Quantitative analysis of centriole signals and configurations in non-myocytes (**b**) and cardiomyocytes (**c**) from cryosections of P0 (MOCK) and P3 or P6 (SHAM and AR) rat heart ventricles. Results are from three independent animals. ≥200 cardiomyocytes and ≥100 non-myocytes, collectively, from basal and apical regions were analyzed per experimental condition (see also Supplementary Fig. 1). (**d**) Quantitative analysis of cardiomyocytes proximal (within 1 mm) to base or apex/resection with paired centrioles from cryosections of P3 or P6 (SHAM and AR) rat hearts. Data are ± SD. p-values were calculated using two-tailed Student’s t-test.

To determine if apical resection (AR) in postnatal day 1 (P1, 24 h after birth) rats affects centrosome disassembly, the percentage of rat ventricular cardiomyocytes that maintain centrosome integrity (i.e. paired centrioles) *in vivo* was evaluated. Centrioles were considered as paired if centrioles proximal to the nuclear envelope were within 2 microns of one-another (Fig. 1a)^11^. As a negative-control for centrosome disassembly, the percentage of ventricular cardiac non-myocytes (which do not undergo developmentally-regulated centrosome disassembly)^9^ with paired centrioles was evaluated in P1 MOCK (no surgery) as well as P3 and P6 SHAM and AR hearts. No indication of developmentally-regulated centrosome disassembly was observed in non-myocytes (Fig. 1b). In contrast, the percentage of cardiomyocytes with paired centrioles progressively declined as development progressed in both SHAM and AR hearts, indicating that AR does not perturb developmentally-regulated centrosome disassembly (Fig. 1c). Moreover, AR resulted in a statistically significant (p = 0.0213) reduction in the percentage of cardiomyocytes with paired centrioles at P3 (Fig. 1c). To further evaluate the centrosome-related terminally differentiated phenotype, the effect of AR on localization of Pericentrin, which accumulates at the nuclear envelope during terminal differentiation, was also evaluated. AR did not negatively affect Pericentrin accumulation at the nuclear envelope, and, further, resulted in an increase in the percentage of Pericentrin-positive cardiomyocyte nuclei at P6 (Supplementary Fig. 1). These results indicate that AR does not inhibit, delay, or reverse the developmental process regulating centrosome disassembly in cardiomyocytes but rather accelerates it. Spatial analysis of centrosome disassembly in P3 hearts showed that AR had little effect on centrosome disassembly proximal to the apex/injury zone (Fig. 1d). This suggests that while injury temporally accelerates centrosome disassembly, this effect is either suppressed or non-existent proximal to the injury zone.

### Apical resection does not perturb cardiomyocyte binucleation

**Figure 2:**
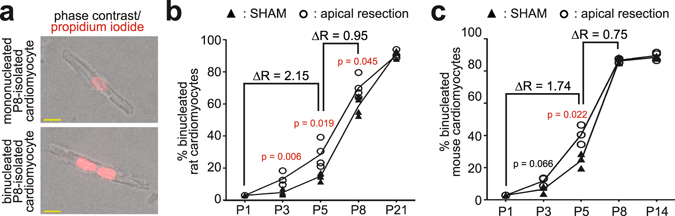
Analysis of cardiomyocyte binucleation in AR hearts. (**a**) Representative images of mononucleated and binucleated P8-isolated rat cardiomyocytes. Nuclei were counterstained with propidium iodide. Yellow scale bars: 10 μm. (**b**,**c**) Percent binucleated cardiomyocytes at different time points after AR and SHAM in P1 rat (n = 4 for each time point, (**b**) and mouse (n = 3 for each time point, (**c**), see also Supplementary Fig. 2) hearts. p-values were calculated using two-tailed Student’s t-test comparing AR and SHAM. Inflection points reflect mean values. ΔR = fold difference in the rate of binucleation of AR- compared to SHAM-operated hearts. P1 data point for rat is from Li *et al*. (JMCC, 1996) and for mouse from Soonpaa *et al*. (AJP, 1996). p-values considered here significant (p < 0.05) are indicated in red.

To determine if injury affects binucleation, ventricular cardiomyocytes were isolated at P3, P5, and P8 from AR and SHAM rat hearts. Cardiomyocytes were identified by phase-contrast imaging of sarcomeres and the number of nuclei was determined by propidium iodide staining (Fig. 2a). The percentage of binucleated cardiomyocytes throughout neonatal development in the SHAM groups was similar to those previously described^4^. Relative to SHAM, AR resulted in an increase in the percentage of binucleated cardiomyocytes at P3, P5, and P8 (Fig. 2b), but not at P14. Further, relative to SHAM, AR induced a nearly 2-fold increase in the rate of binucleation during early neonatal development (P1 to P5), indicating that injury promotes a transient acceleration in the proportion of cardiomyocytes undergoing binucleation (Fig. 2b). To determine if AR affects the overall frequency of binucleated cardiomyocytes, rat hearts were analyzed at later developmental stages. In P21 rat SHAM and AR hearts, ~90% of cardiomyocytes were binucleated (Fig. 2b). As previous AR studies were performed in mouse hearts, we determined also the binucleation rate in mice after AR (Supplementary Fig. 2). Similar to our data in rats, injury did not perturb binucleation in mice upon AR but rather induced a transient increase in binucleation (Fig. 2c). These results indicate that injury does not inhibit the developmental process of cardiomyocyte binucleation but rather, transiently, promotes it.

### Apical resection increases the frequency of cardiomyocytes in mitosis

**Figure 3:**
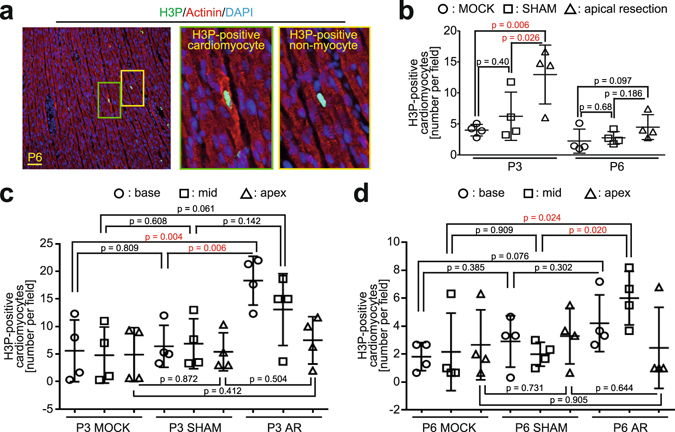
Analysis of H3P-positive cardiomyocytes in AR rat hearts. (**a**) Representative immunofluorescence images of a P6 H3P-positive cardiomyocyte and non-myocyte (scale bar: 50 µm). Red: cardiomyocytes (sarcomeric-α-actinin); green: cells in mitosis (H3P); blue: nuclei (DAPI). Scale bar: 50 µm (see also Supplementary Fig. 3). (**b**) Quantification of cardiomyocytes in mitosis at P3 and P6 based on anti-H3P and anti-sarcomeric-α-actinin staining. Nuclei were counterstained with DAPI. Data are ± SD. n = 4 for MOCK, AR, and SHAM at each time point. (**c**,**d**) Quantification of H3P-positive cardiomyocytes at P3 (**c**) and P6 (**d**) in apical, mid, and base zones. Data are ± SD. n = 4 for MOCK, AR, and SHAM at each time point. p-values have been calculated using Fisher’s LSD test after one-way Anova.

A transient burst in the rate of cardiomyocytes undergoing binucleation should be reflected by a temporal increase of cardiomyocytes entering their terminal cell cycle. To determine this, we compared the number of cardiomyocytes in mitosis at P3 and P6 in MOCK, SHAM, and AR hearts. Cardiomyocytes in mitosis were identified as H3P-positive cells exhibiting a disassembled sarcomeric apparatus (Fig. 3a and Supplementary Fig. 3). No difference in the number of cardiomyocytes in mitosis between MOCK (i.e. no surgery) and SHAM hearts at P3 and at P6 was observed (Fig. 3b), indicating that the SHAM surgical procedure did not influence cardiomyocyte cell cycle activity. In contrast, compared to both MOCK and SHAM hearts, AR resulted in a statistically significant and nearly 2-fold increase in the mean number of cardiomyocytes in mitosis at P3 (~5% to ~13% per field) (Fig. 3b). This 2-fold increase was not apparent at P6. Thus, consistent with a transient burst in the rate of binucleation, AR promotes a transient burst in cardiomyocyte cell cycle activity. Spatial analysis of the number of cardiomyocytes in mitosis in P3 and P6 hearts showed that AR had little effect on cardiomyocyte cell cycle activity proximal to the apex/injury zone (Fig. 3c,d). This suggests that while injury temporally increases cardiomyocyte cell cycle activity, this effect is either suppressed or non-existent proximal to the injury zone.

### Apical resection does not result in cardiomyocyte cytokinesis associated with cell division

**Figure 4:**
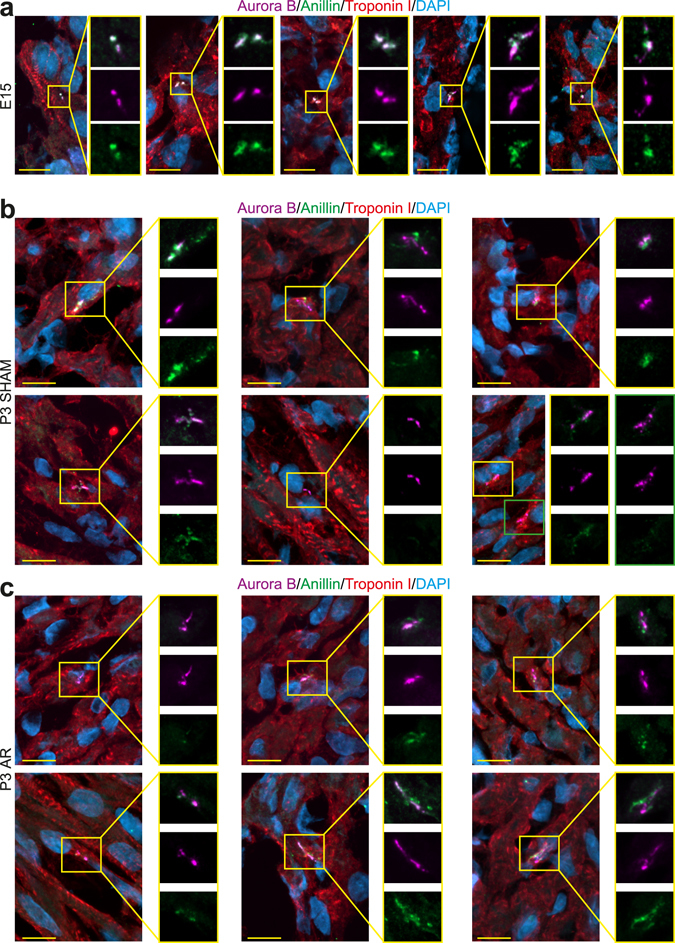
Characterization of cardiomyocyte cytokinesis *in vivo*. Representative images of rat heart sections stained for cytokinesis proteins Aurora B (purple) and Anillin (green) and cardiomyocytes (Troponin I, red). Nuclei were visualized with DAPI (blue). (**a**–**c**) Representative images of cardiomyocytes undergoing midbody formation in hearts from E15 (**a**) cytokinesis results in cell division), P3 SHAM (**b**) cytokinesis results in binucleation) and P3 post-AR (**c**) rats. Scale bars: 10 µm.

Although our data indicate that transient increases in cardiomyocyte binucleation can account for a significant fraction of AR-induced increases in cardiomyocyte cell cycle activity, it remains unclear to what degree AR can promote division. To address this, we evaluated cardiomyocyte cytokinesis, which can either result in successful abscission (i.e. division) or abscission failure (i.e. binucleation). It has previously been shown in P3-isolated neonatal rat cardiomyocytes undergoing binucleation that Anillin, a component of the cleavage furrow required for successful cellular abscission, is mislocalized during midbody formation^5^. To evaluate if AR results in cardiomyocytes undergoing successful abscission, we analyzed Anillin localization relative to Aurora B, a commonly used marker to identify cells undergoing cytokinesis (note, Aurora B at the midbody alone cannot predict if a cell will successfully complete abscission)^5^. A “normal cytokinesis” (i.e. one that results in abscission/division) was scored as one where Anillin is localized at the Aurora B-defined stembody and/or was co-localized with Aurora B. An “abnormal cytokinesis” (i.e. one that results in abscission failure/binucleation) was scored as one where Anillin a) lacked any stembody co-localization, b) lacked any AuroraB co-localization and/or c) exhibited a diffused/de-condensed pattern. As positive controls for these staining patterns *in vivo*, we first evaluated cryosections from E15 and P3 hearts, where cardiomyocytes entering cytokinesis undergo abscission and binucleation, respectively. We found that cardiomyocytes from E15 rat hearts exhibited a “normal cytokinesis”, consistent with their ability to complete abscission (Fig. 4a). Cardiomyocytes from P3 rat SHAM hearts exhibited an “abnormal cytokinesis”, consistent with their inability to undergo abscission (Fig. 4b). To rule out the possibility that developmental age of the heart results in different Anillin staining patterns, irrespective of whether a cytokinesis is normal or abnormal, we analyzed P1 SHAM hearts which consist of both binucleating and dividing cardiomyocytes^4, 12^. If the unique E15 and P3 Anillin staining patterns are a function of age, then only a single Anillin staining pattern should be observed. However, if the Anillin staining pattern is a function of whether a cardiomyocyte binucleates or divides, then both Anillin staining patterns should be observed. Consistent with the occurrence of proliferation and binucleation, we found that a subset of cardiomyocytes from P1 SHAM hearts exhibited “normal” (Supplementary Fig. 4a) and a subset that exhibited “abnormal cytokinesis” (Supplementary Fig. 4b). These data suggest that Anillin staining patterns can be used as an indicator of whether a cardiomyocyte entering cytokinesis divides or binucleates. In order to determine whether AR induced cardiomyocyte proliferation we analyzed the Anillin staining pattern in P3 hearts. We were unable to identify any cardiomyocytes undergoing midbody formation which exhibited a “normal cytokinesis” (Fig. 4c). These results indicate that binucleation, and not division, is the more likely outcome of AR-induced increases in cardiomyocyte cell cycle activity.

### Apical resection in rats results in persistent scarring and decreased wall motion

**Figure 5:**
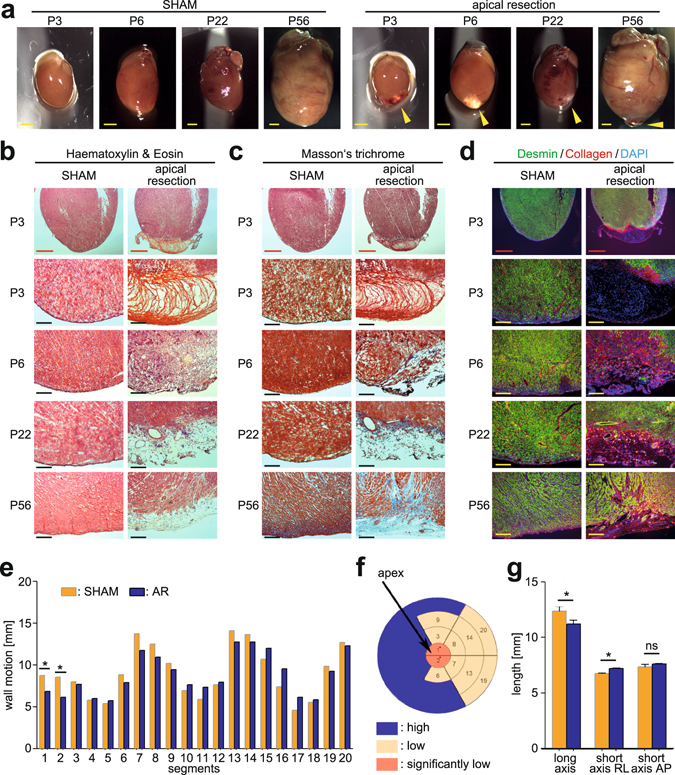
Characterization of AR rat hearts. (**a**) Representative stereomicroscopic images of SHAM- and AR-operated rat hearts at different postnatal time points. Arrow heads indicate myocardial scar. Scale bars: 1 mm. (**b**–**d**) Representative images of Hematoxylin & Eosin staining (**b**), Masson’s trichrome staining (**c**), and immunofluorescence analysis of non-myocytes (Collagen I/Desmin/DAPI) (**d**) of SHAM- and AR-operated hearts at different postnatal time points. P3–P22 (n = 4–6) and P56 (n = 3) for each group. Red scale bars: 1 mm. Black/yellow scale bars: 200 µm. (**e**) The mean wall motion of each heart segment was compared between SHAM and AR hearts (n = 3). (**f**) Summary of wall motion depicting heart segments in AR hearts versus those of SHAM demonstrates defects in the apex region. (**g**) Quantitation of heart axes by PET scanning (n = 3). Right-left (RL). Anterior-posterior (AP). Data are ± SEM. *p < 0.05.

Analysis of gross morphology showed that AR rat hearts exhibited persistent scarring at P56 in the apical region (Fig. 5a). H&E staining showed that P3 hearts, and to a lesser extent P6 hearts, exhibited a blood clot that was absent by P22 (Fig. 5b). Masson´s trichrome staining showed fibrotic deposition in the apical region at P22 (Fig. 5c). Immunofluorescence analysis showed that Desmin/Collagen I-positive non-myocytes dominated the fibrotic zone (Fig. 5d). Scarring was reflected in reduced wall motion of the apex as assessed 26 days after AR using F-18-fluorodeoxyglucose positron emission tomography (FDG-PET) (Fig. 5e,f). Quantification of the heart axes using FDG-PET showed a 10% shortening of the long axis in AR hearts (Fig. 5g), which is in agreement with our previous results in mouse^13^, reflecting minimal apex outgrowth. Collectively, these observations indicate that AR exposing the left ventricular chamber does not result in complete regeneration in terms of scar removal or restoring wall motion.

## Discussion

Based on the analysis of centrosome disassembly and binucleation, our results indicate that AR in newborn hearts does not inhibit, delay, or reverse but rather enhances the rate of cardiomyocyte terminal differentiation. These data are surprising as several studies have suggested that AR results in regeneration through global cardiomyocyte proliferation^10, 14, 15^.

It is important to note that our data does not directly address to what degree AR promotes cardiomyocyte proliferation. Indeed, the consensus in the field is that AR induces cardiomyocyte proliferation (after P3). However, proliferation has not been unambiguously proven in the neonatal injury model. Methods used to detect proliferation after neonatal injury have been limited to markers for S-phase (i.e. EdU-incorporation), mitosis (H3P expression), and cytokinesis (Aurora B midbody localization). However, these markers do not delineate proliferation vs. binucleation. Cardiomyocytes that binucleate also progress through S-phase^4^ and mitosis^5^. Further, both dividing and binucleating cardiomyocytes, exhibit contractile ring formation^6^ as well as aurora B kinase midbody localization^5^. Thus, markers for S-phase, mitosis, and cytokinesis are not evidence that proliferation has occurred.

In an attempt to address the issue to what degree cardiomyocytes divide after AR in rats, we evaluated Anillin staining patterns relative to Aurora B. This approach is more informative than Aurora B alone for 3 reasons: 1) Aurora B alone cannot detect whether a cardiomyocyte will divide or binucleate, 2) detecting true Aurora B signals *in vivo* can be difficult, due to artifacts arising during the staining procedure, potentially leading to false-positives (i.e. co-localization of Anillin and Aurora B reduces the likelihood of scoring false-positives) and 3) Anillin and Aurora B co-staining provides information as to whether the cardiomyocyte cytokinesis is “normal” or “abnormal”. We were unable to identify any cardiomyocytes undergoing a “normal cytokinesis” in P3 AR hearts. While we cannot rule-out the possibility that an “abnormal cytokinesis” can still result in division, the conservative deduction is that “abnormal cytokinesis” results in abscission failure (i.e. binucleation). Thus, our data weigh towards binucleation, and not proliferation, as being the more prominent outcome of cardiomyocyte cell cycle activity after AR.

It is worth noting that diffused Anillin staining patterns observed in this study in P3 hearts are less prominent than that observed in P3-isolated rat ventricular cardiomyocytes^5^. This difference in the extent of Anillin diffusion is most likely due to the difference of *in vitro* and *in vivo* environments (2- vs 3- dimensional) under which cytokinesis is occurring. For example, isolated cardiomyocytes *in vitro* are spread resulting in a more extended Anillin pattern.

Although our study does not address directly how much cardiomyocyte proliferation occurs in response to AR, our data are nevertheless incompatible with significant levels of cardiomyocyte proliferation after AR. This is concluded based on the following: 1) It is well established that murine cardiomyocytes do not proliferate after ~P3^4, 12^. Thus, in SHAM, the observed number of H3P events at ~P3 (and beyond) reflects binucleation. 2) We observed that AR induces a ~2-fold increase in the rate of binucleation from P1–P5. If (extensive) proliferation was occurring along-side binucleation during the same period, in order to maintain the observed percentage of binucleated cardiomyocytes in P5 AR hearts, the number of H3P events in P3 AR vs. SHAM hearts would have to be significantly greater than a 2-fold increase (Supplementary Fig. 5), which we did not observe. 3) Further, we found that AR does not induce an increase in the rate of binucleation relative to SHAM from P5–P8. If proliferation were occurring along-side of binucleation, then one would predict an increase in H3P events at P6 in AR vs. SHAM, which we did not observe. 4) We did not find evidence of cardiomyocytes in P3 AR hearts undergoing “normal cytokinesis”.

Porrello and coworkers excluded the possibility of neomyogenesis based on stem cells^10^. Our data argue against extensive cardiomyocyte proliferation post P3. However, it has to be noted that our argumentation holds only true for proliferation of mononucleated cardiomyocytes. Thus, it might be possible that neonatal injury induces proliferation of binucleated cardiomyocytes, which would be in agreement with the currently available data. However, there is limited evidence in the literature that binucleated cardiomyocytes can divide under non-modified conditions (e.g. forced oncogene expression^16^). Collectively, the current literature together with our data questions the widely-held consensus of neomyogenesis based on endogenous cardiomyocyte proliferation.

To evaluate regeneration, besides H3P-based analysis (for neomyogenesis), neonatal cardiac injury studies often evaluate heart weight (HW) and degree of scar formation. The mouse heart weighs 11.2 mg at P2 and 67 mg at P19^12^. Assuming a normal course of neonatal development with no compensatory hypertrophy or compensatory proliferation, a 10% (by weight) AR at P2 will result in a P19 heart that weighs 64.8 mg (or 0.967 relative to sham). This value may be statistically difficult to observe. Indeed, Bryant and coworkers were unable to identify statistical significance in HW between AR and SHAM at P21^14^.

Scar removal is a hallmark of zebrafish regeneration. Yet, there are a number of studies that show that this does not occur after AR in neonatal mice^14, 15, 17–19^. Further, it has been observed that smaller injuries produce less “scar infiltration” (i.e. the amount of cardiac tissue present between the ventricle and scar)^14^. This led to the conclusion that smaller injuries are met with greater (proliferation-based) regeneration. However, it is well known that i) binucleation occurs during neonatal development, ii) binucleation results from failed cell division, iii) a cell that fails to divide is nearly twice as large as its parent cell. Thus, the more mononucleated cardiomyocytes proximal to the apex at P1 after AR, the more cardiomyocytes are present to undergo binucleation and increase tissue mass by P21. Thus, binucleation (or, developmental neomyogensis) alone can explain the observation by Bryant and coworkers that smaller resections result in smaller “scar infiltration”.

Why the regenerative response of newborn mouse hearts is not proportional to injury size, with small injuries having greater regeneration potential than large injuries^14^, remains unknown. Our results suggest a cellular mechanism for this phenomenon (assuming that binucleated cardiomyocytes cannot proliferate). That is, as injury does not inhibit the developmental timing that renders cardiomyocytes post-mitotic, the fraction of proliferating mononucleated cardiomyocytes is progressively decreasing, thereby limiting the maximum extent of neomyogenesis, regardless of injury size.

What accounts for a transient burst in cardiomyocyte binucleation remains unclear. It is well known that several factors such as hypoxia, glucocorticoids, hypertension, and hypertrophic stimuli increase the fraction, and or degree of, cardiomyocyte multinucleation^20, 21^. Thus, binucleation might be a physiological response to neonatal injury, similarly as an increase in cell cycle activity and in ploidy has been observed in heart failure patients^22^. With regards to mechanism underlying the transient burst in binucleation, it has previously been shown that AR induces inflammation, and that inflammation is required for AR-induced increase in H3P-positive cardiomyocytes^18^. The mitogenic properties of a number of inflammatory cytokines have been well documented, and these factors are also short-lived. Thus, as our results indicate that AR-induced increases in H3P-positive cardiomyocytes mainly equates binucleation, and not proliferation, it is plausible that AR-induced inflammation provides a short-lived stimulus which temporarily accelerates cardiomyocytes to enter their terminal cell cycle, thus accounting for the transient burst in cardiomyocyte binucleation.

While it is generally accepted that AR in new-born mice increases the number of mitotic cardiomyocytes, Andersen and coworkers have reported that AR in mice does not promote an increase in cardiomyocyte cell cycle activity based on EdU incorporation^17^. In the EdU labelling experiments, mice were injected a single time with EdU 1 day after surgery and the number of EdU-positive cardiomyocytes was determined at P7. In contrast, Porrello and coworkers performed 3 injections, at P1, P7, and P14, and hearts were analyzed at P21^10^. Given that the half-life of EdU is less than 24 h and cell cycle activity was measured between ~P1 to ~P2, Andersen and coworkers may have failed to detect an increase in cardiomyocyte cell cycle activity as observed in many other studies, including the current study, at later time points.

We conclude that a regenerative program might exist that promotes cardiac regeneration based on cardiomyocyte proliferation until P3. Yet, our data suggest that it is quickly curtailed by developmental mechanisms that render cardiomyocytes post-mitotic. This would explain why the regenerative response of newborn mouse hearts is not proportional to injury size and is absent in P7-old mice.

## Methods

### Animals

#### Rats

Pregnant Sprague Dawley rats were purchased from Taconic Europe. Briefly, animals were housed in plastic cages with a 12/12 hours light/dark cycle, and fed ad libitum with a chow appropriate for pregnant rodents. To ensure robustness of animal experiments performed herein, both SHAM- and AR-operated rats were enclosed in all used litters. For the preparation of E15 heart sections, pregnant Sprague Dawley rats were purchased from Charles River and sacrificed. All rat experiments conform with the Guide for the Care and Use of Laboratory Animals published by the Directive 2010/63/EU of the European Parliament and were approved by the Danish Council for Supervision with Experimental Animals (#2011/561-1966) or by the local Animal Ethics Committee of Erlangen in accordance to governmental and international guidelines on animal experimentation (protocol TS—9/2016 Nephropatho).

#### Mice

E17 Pregnant CD-1 (ICR) mice were purchased from Charles River and housed at the animal facility in Boston Children’s Hospital. P1 pups were subjected to AR. All animal experiments were performed in accordance with the relevant guidelines and regulations and were approved by the Institutional Animal Care and Use Committee (IACUC) at Boston Children’s Hospital.

### Apex Resection

#### Rats

AR in P1 rats (n = 51) was performed as previously described^17^. Briefly, P1 neonates were anaesthetized by hypothermia for approximately 6 min. Anaesthetized rats were given 0.05 ml 0.9% NaCl twice subcutaneously to avoid dehydration. Using stereomicroscopy, left parasternal and horizontal skin incision was performed followed by thoracotomy using blunt dissection. To avoid that the resected apex tissue goes into the thoracic cavity, microsurgical forceps were utilized to gently fix this tissue. An iridectomy scissor was used to resect the apex until ventricle chamber exposure was achieved as previous described by Porrello and coworkers^10^. Blood clotting sealed the heart and omitted the use of suturing. AR-resected animals were then removed from ice and the thoracic wall and skin incision were closed. Animals quickly recovered from anesthetics under a heat lamp, and were returned to their mother as soon as natural movements and a red/pink complexion were achieved. SHAM animals underwent the exact same procedure though without disturbing or resecting the apex of the heart. The obtained AR rat survival rate was 90.3 ± 5.3% which is higher than the 70% reported by Porrello and coworkers^10^. These data indicate that we have not resected more tissue than reported previously. Rats were sacrificed at indicated time points by cervical dislocation. Hearts were then carefully dissected avoiding any further damage.

#### Mice

AR in P1 mice (n = 39) was performed using published techniques^23^. Briefly, P1 neonates received subcutaneous injections of 0.1% bupivacaine, placed inside a protective sleeve, and submerged into an ice-water bath until they were non-responsive to the paw reflex. Using stereomicroscopy, a ventrolateral thoracotomy was performed between the fourth and fifth ribs, the pericardium was removed, and the heart was exposed. The cardiac apex was serially resected using a pair of angled microscissors until the left ventricular cavity was exposed (as evidenced by free flowing blood when the heart was squeezed). SHAM consisted of opening the chest and removing the pericardium. The chest and skin were closed with 8–0 Prolene sutures. Pups recovered under a heating lamp, placed on a warming blanket until they became responsive, rubbed with bedding and returned to their mothers. At selected time points, P1–P7, mice were euthanized by decapitation. Mice at P8 and older were euthanized by isoflurane. Hearts were carefully removed from the thoracic cavity, immediately placed into ice-cold cardioplegic solution, blood removed from the ventricular cavities, and the hearts placed into phosphate buffered formalin for 24 h at 4 °C and then placed in phosphate buffered saline (PBS) until further analysis.

### Immunostaining

For immunostaining, 10 µm-thick cryosections were fixed in 3.7% formalin (Sigma) for 10 min at room temperature (RT). Tissue was blocked utilizing 3% bovine serum albumin (BSA) (Sigma) diluted in PBS. Antibody staining was performed at RT for 1 h if not stated otherwise. Primary antibodies for analysis of mitotic cardiomyocytes: mouse anti-sarcomeric-α-actinin (1:250, Abcam, Cambridge), rabbit anti-phospho-histone H3-Serine 10 (H3P, 1:500, Santa Cruz Biotechnology). Primary antibodies for analysis of centrosome integrity: mouse anti- γ-tubulin (1:500, Sigma), goat anti-Nkx2.5 (1:200, Santa Cruz Biotechnology). Primary antibodies for analysis of translocation of Pericentrin (4 °C, overnight): goat anti-Nkx2.5 (1:1000, Santa Cruz Biotechnology), mouse anti-caveolin 3(BD Transduction Laboratories), rabbit anti-Pericentrin (1:1000, BioLegend). Primary antibodies for analysis of cytokinesis (4 °C, overnight): mouse anti-Aurora B (AIM1, 1:500, BD Transduction Laboratories), rabbit anti-Anillin (1:100, gift by C. Field), goat anti-Troponin I (1:500, Abcam). Primary immune complexes were detected with ALEXA 488-, ALEXA 594- or ALEXA 647-conjugated antibodies (1:500, Life Technologies). All antibodies were diluted in 3% BSA. DNA was stained with 0.5 µg/ml DAPI (4′,6′-diamidino-2-phenylindole) (Sigma). For analysis of collagen/desmin deposition in rat hearts, paraffin tissue sections (7 µm) were deparaffinized as described previously^17^. Antigen retrieval was performed by boiling sections in Tris-EGTA (pH 9). Sections were then blocked 10 min in 2% BSA diluted in tris buffered saline (TBS). Primary antibodies (rabbit anti-collagen I (1:100, Abcam) and goat anti-desmin (1:50, Santa Cruz Biotechnology) diluted in 1% BSA/TBS were applied overnight at 4 °C. Primary immune complexes were detected with ALEXA 555- or 488-conjugated antibodies (1:200, Molecular Probes). Samples were mounted in medium containing DAPI (Vectashield, Vector Lab).

### Quantitation of rat cardiomyocyte mitosis

The number of mitotic cardiomyocytes from AR and SHAM P3 and P6 rat hearts were scored in 3 different zones (i.e. the apical, mid, and base zones) and 3 different fields (700 × 550 µm, the number of Nkx2.5 nuclei per field was comparable) per zone. For each condition and time point 4 hearts were analyzed.

### Quantitation of centrosome disassembly in rat cardiomyocytes

The percentage of rat cardiomyocytes from AR and SHAM P3 and P6 rat hearts were scored in 2 different zones (i.e. the apical and base zones) and 3 different fields (700 × 550 µm) per zone. Apical zone was defined as tissue within 1 mm from apex, and base zone was defined as tissue within 1 mm from the base. For each condition and time point 3 hearts were analyzed. Note, that there are two centrosome configurations: i) paired, indicates centrosome integrity. ii) split, indicates centrosome disassembly, which is consistent with loss of proliferative potential. The singlet “configuration” is indicative of either split (where the other centriole is out of the section plane) or paired (where the two centrioles are positioned atop one another relative to the viewing plane). The “no signal” configuration is indicative that neither centriole is present in the section plane.

### Determination of cardiomyocyte nucleation

Neonatal hearts were fixed in 3.7% formalin at 4 °C for 24 h. Hearts were then minced, and cardiomyocytes dissociated from heart tissue using 0.5 to 1.5 M KOH on a table-top shaker for 24 h at room temperature. Nuclei were counter-stained with propidium iodide (0.1 mg/ml) for 10 min. Cells/tissue were then centrifuged at 300 × g, washed once with PBS, and briefly vortexed. Cardiomyocytes were distinguished from non-myocytes based on their characteristic elongated form and presence of striations (as determined by phase contrast imaging).

### Imaging

Immunofluorescence images of cryosectioned tissue for rat cardiomyocyte mitosis analysis: Images were captured on a Keyence BZ9000 Fluorescence Microscope (Keyence, Osaka, Japan) or a LSM800 confocal laser scanning microscope (ZEISS). Gross morphological examinations of rat hearts were performed using LeicaM80 with a LeicaIC80HD camera whereas immunofluorescence and histology examinations were performed with a Leica DMI4000B Cool Fluo Package instrument equipped with a Leica DFC340 FX Digital Cam and a Leica DFC 300 FX Digital cam. Images of mouse heart gross morphology were acquired with an Olympus Stereoscope (SZX16, Olympus) equipped with a QIClick CCD color camera (QImaging). Exposure (camera settings) and picture processing (slight adjustment of contrast/brightness and color balance by using Photoshop) were applied equally to sample sections. Images were arranged with ImageJ (Public Domain) and Adobe Illustrator (Adobe, San Jose, CA, USA).

### Analysis of Anillin localization in rat cardiomyocyte cytokinesis

Anillin staining pattern in cytokinesis was assessed in E15, P1, P3 SHAM and P3 AR rat heart ventricles. Cardiomyocytes were identified using Troponin I. Cytokinesis was defined by Aurora B signal localizing to the midbody arms leaving a blank spot in between which corresponds to the stembody^24^. Anillin pattern was scored “normal” if it localized at the stembody and/or concentrated around the Aurora B signal. “Abnormal” Anillin pattern refers to a cloudy, de-condensed Anillin signal and/or a delocalization of Anillin from the Aurora B signal. 30 cytokinesis from 3 hearts were scored per condition and time point.

### F-18-fluorodeoxyglucose positron emission tomography (FDG-PET)

To assess cardiac function, animals underwent FDG-PET imaging as described recently^13^, but with minor adjustment for animal size. Briefly, rats were anesthetized and injected with FDG (43.3 ± 2.1 MBq) before FDG-PET imaging using a small animal PET scanner (INVEON, Siemens pre-clinical solutions, Knoxville, TN, US). FDG-PET images were analyzed in a blinded manner by a medical physicist experienced in using the QGS software (Cedars-Sinai Medical Center, Los Angeles, CA, USA). This software allows for automatic processing with only alignment of the heart axes as a variable thus minimizing the bias of the analysis.

### Statistical analysis

Statistical analysis was conducted using Matlab R2014b. Data for mitotic cardiomyocytes consisted of four independent experiments per condition per time point. For analysis of the number of mitotic cardiomyocytes, one-way ANOVA followed by Fisher’s LSD test was employed. For analysis of the percentage of binucleated cardiomyocytes, cardiomyocytes with paired centrioles, and Pericentrin-positive cardiomyocyte nuclei between SHAM and AR hearts at a given time point, a two-tailed Student’s *t* test was performed. Data for percentage of binucleated rat cardiomyocytes consisted of four independent experiments per condition per time point. Data for percentage of binucleated mouse cardiomyocytes consisted of three independent experiments per condition per time point. The binucleation rate R between two time points was calculated as the coefficient of the least-square linear regression fit; and ΔR is the ratio between the binucleation rate in AR versus SHAM.

### Data Availability

No datasets were generated or analyzed during the current study.

## Electronic supplementary material

Supplementary Information
