# Supplementary material for: Cardiac injury of the newborn mammalian heart accelerates cardiomyocyte terminal differentiation
Source: Sci Rep. 2017 Aug 21;7:8362. doi: 10.1038/s41598-017-08947-2 (PMC5567176; doi:10.1038/s41598-017-08947-2)

Supplementary Information for

Cardiac injury of the newborn mammalian heart accelerates cardiomyocyte terminal differentiation

David C. Zebrowski, Charlotte H. Jensen, Robert Becker, Fulvia Ferrazzi, Christina Baun, Svend Hvidsten, Søren P. Sheikh, Brian D. Polizzotti, Ditte C. Andersen, Felix B. Engel

Content:

1) Supplementary Figure Legends
2) Supplementary Figure 1
3) Supplementary Figure 2
4) Supplementary Figure 3
5) Supplementary Figure 4
6) Supplementary Figure 5

Supplementary Figure Legends

Supplementary Figure 1: Effect of AR on nuclear localization of pericentrin. (**a**, **b**) Representative images of cryosections of P3 rat heart ventricles. red/cyan: cardiomyocytes (nuclei: Nkx2.5; cytoplasmic membrane: caveolin 3), green: pericentrin (pericentriolar matrix/centrosome), blue: nuclei (DAPI). Dotted circle: vessel. Arrows: examples of cardiomyocytes with pericentrin localized to the nucleus. Scale bars: a: 25 µm, b: 10 µm. (**a**) Note: 1: Non-myocyte with pericentrin localized to its centrosome (asterisk). 2: Cardiomoyocyte with pericentrin localized to its centrosome. 3: Cardiomyocyte with pericentrin localized to the nucleus (arrow). (**c**) Quantitative analysis of cardiomyocytes proximal (within 1 mm) to base or apex/resection with nuclear pericentrin signal from cryosections of P3 or P6 (SHAM and AR) rat hearts. Data are ± SD. p-values were calculated using two-tailed Student's t-test.

Supplementary Figure 2: Gross morphology of AR mouse hearts. Representative stereomicroscopic images of SHAM- and AR-operated mouse hearts at different postnatal time points. Arrow heads indicate myocardial scar. Scale bars: 1 mm.

Supplementary Figure 3: Analysis of H3P-positive cardiomyocytes in AR rat hearts. Representative immunofluorescence images of sham- and AR-operated P6 rat hearts (scale bars: 1 mm). Red: cardiomyocytes (sarcomeric-α-actinin); green: mitotic cells (H3P); blue: nuclei (DAPI).

Supplementary Figure 4: Characterization of P1 cardiomyocyte cytokinesis *in vivo*. Representative images of rat heart sections stained for cytokinesis proteins Aurora B (purple) and Anillin (green) and cardiomyocytes (Troponin I, red). Nuclei were visualized with DAPI (blue). (**a**) Representative images of P1 SHAM rat cardiomyocytes exhibiting Anillin patterns

associated with “normal” cytokinesis. (**b**) Representative images of P1 SHAM rat cardiomyocytes exhibiting Anillin patterns associated with “abnormal” cytokinesis. Scale bars: 10 µm.

Supplementary Figure 5: Hypothetical model for the amount of H3P events to support proliferation along-side of binucleation after AR. (**a**) SHAM, binucleation with no proliferation. Experimental data: binucleation at P5 = 20%. The number of H3P events at P3 = 1-fold. (**b**) AR, binucleation with no proliferation. Experimental data: binucleation at P5 = 30%. The number of H3P events at P3 = 2-fold. (**c**, **d**) Assumption: all (c) or the additional (d) H3P events after AR indicate proliferation. This would result in a decrease in the binucleation rate which has not been observed. (**e**) Any significant increase in proliferation induced by AR requires a significant increase in binucleation and thus a higher fold-increase in H3P-positive cardiomyocytes than observed. P: postnatal day. Dashed arrows: no cell cycle activity from P3 to P5. Green arrows: mitosis (i.e. H3P event) from P3 to P5. Note, it is known that H3P events post-P3 are strictly followed by binucleation during normal (i.e. SHAM) neonatal heart development.

Zebrowski DC *et al.*, Figure S1

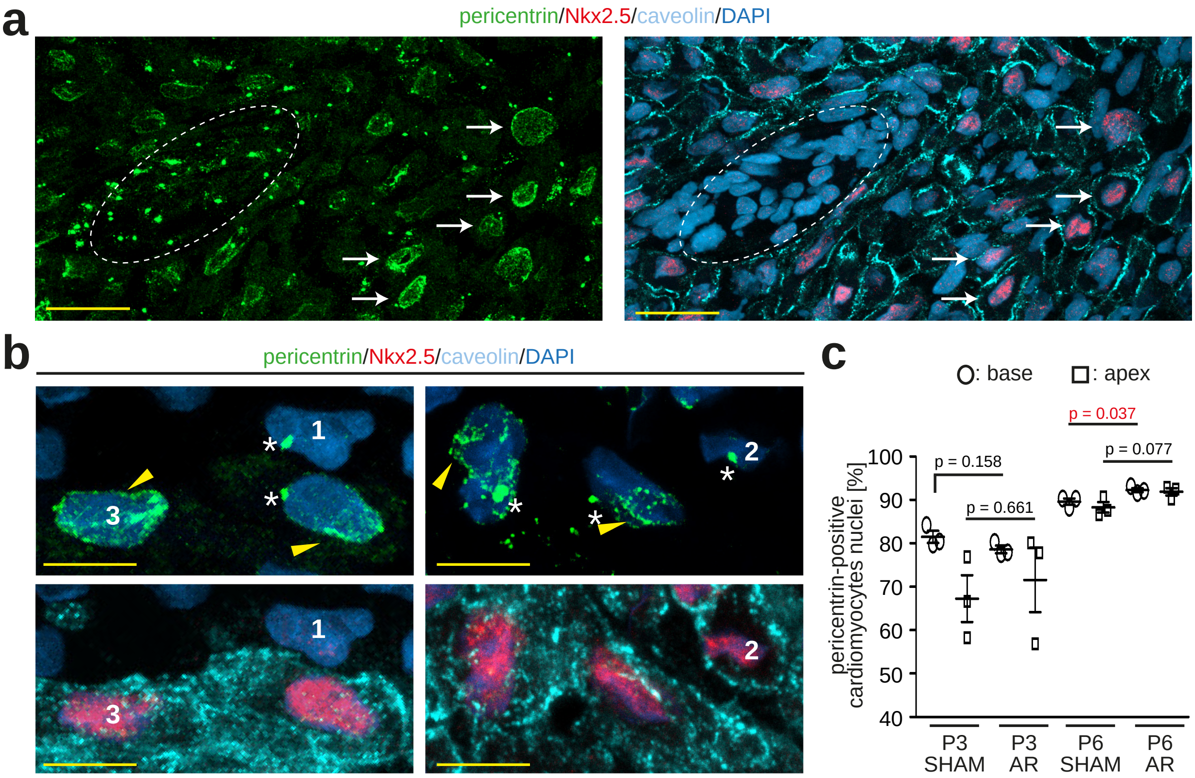

Zebrowski DC *et al.*, Figure S2

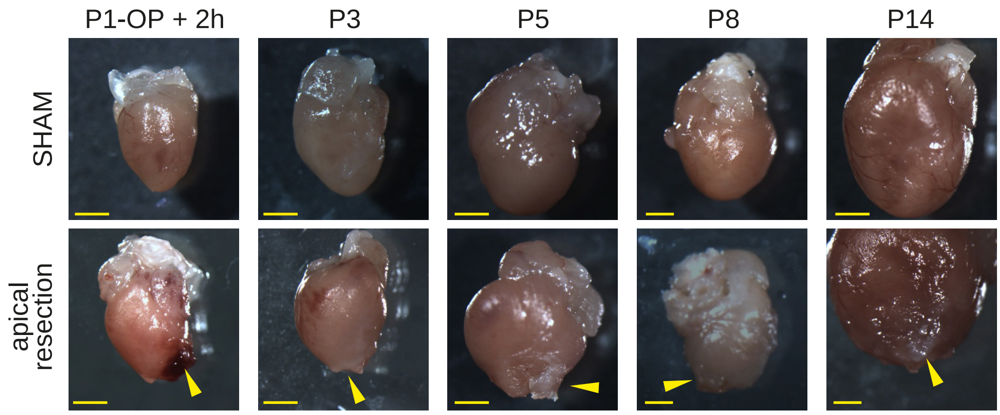

Zebrowski DC et al., Figure S3

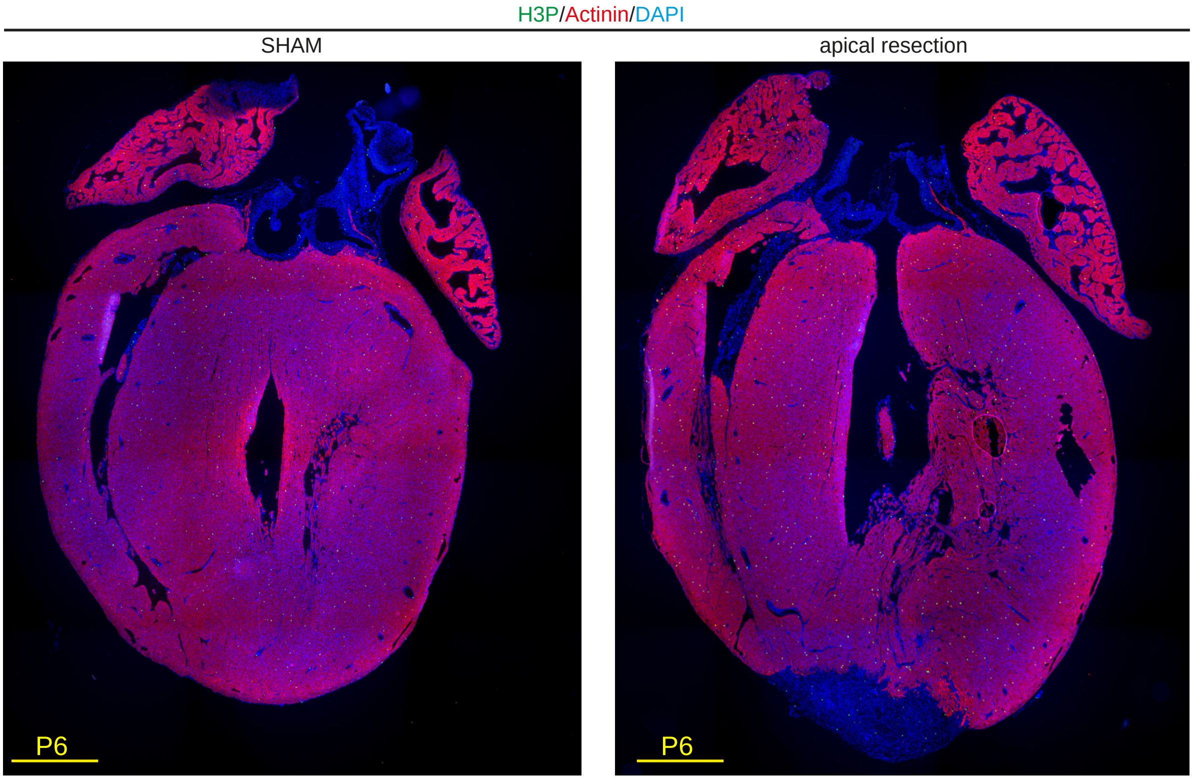

Zebrowski DC *et al.*, Figure S4

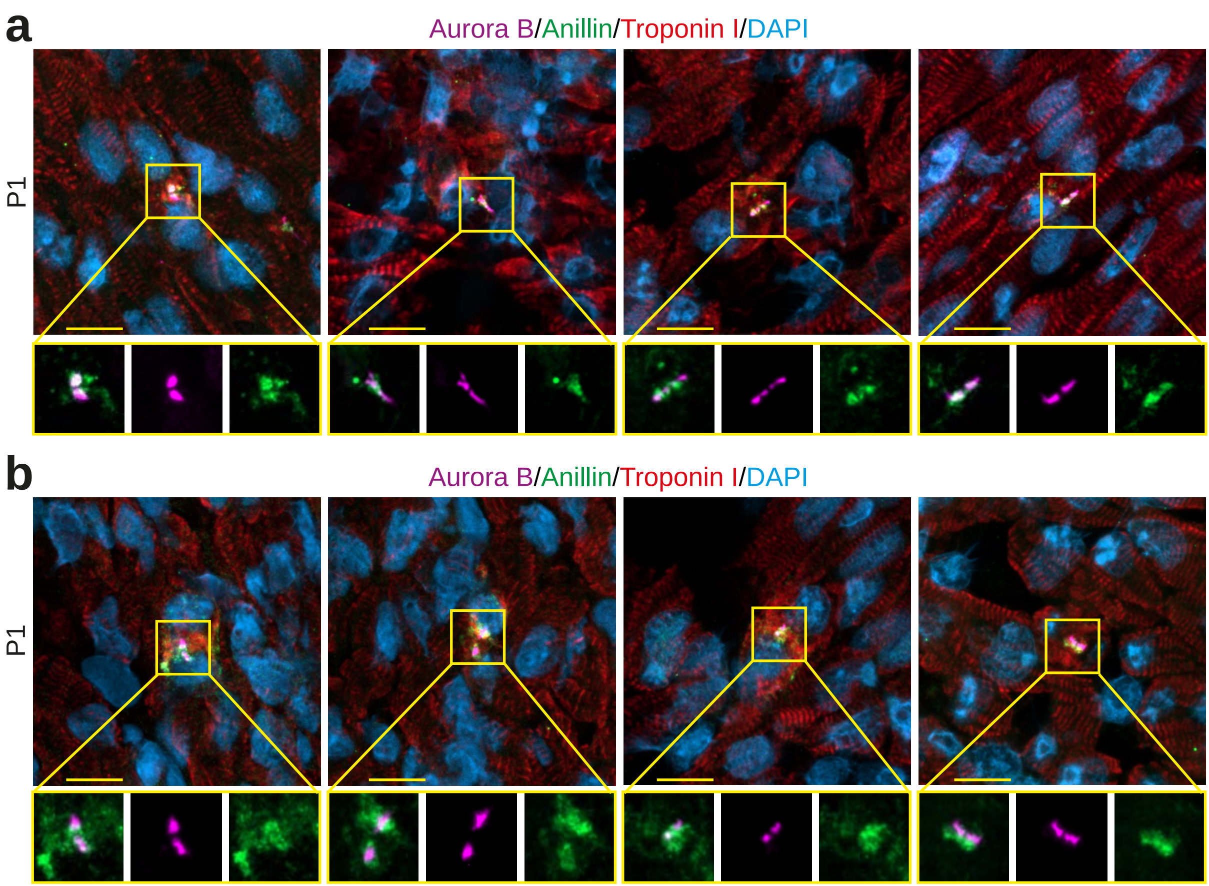

Zebrowski DC *et al.*, Figure S5

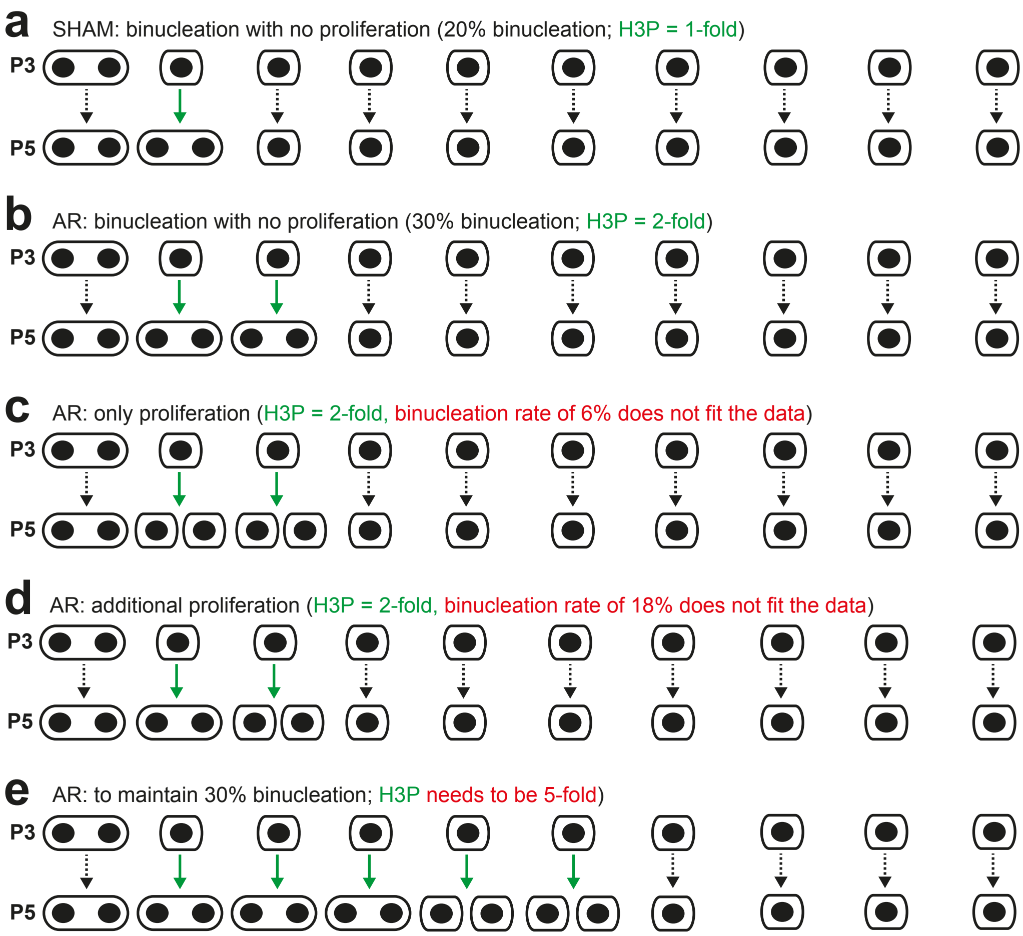

Supplement: Supplementary file 1 — Supplementary Information [file 41598_2017_8947_MOESM1_ESM.pdf]
